# Western Enterprise

**Published:** 1844-07

**Authors:** 


WESTERN ENTERPRISE.

We have been shown a specimen of *A Practical Treatise on Diseases of the Skin*, by Dr. N. Worcester, of Cincinnati, which is to be issued in one volume by Thomas Cowperthwait & Co., Philadelphia, and Desilver & Burr, Cincinnati. The treatise will comprise about 300 pages, and will be accompanied by sixty colored figures. An extensive formulary will also be added. The text is compiled mainly from the works of Alibert, Cazenave, Schedel, Biett, &c., the figures selected from Willan, Bateman, Thompson, Rayer, and Baumes. Of Dr. Worcester's competency to this task, we do not entertain a doubt, and we think he will be doing an essential service to the profession. A treatise, such as he proposes making this, is greatly needed. The specimen before us consists of drawings only, and they look exceeding well.

Dr. Lawson, of Cincinnati, (recently appointed to a professorship in Transylvania University) is republishing, with additions, Hope's *Illustrations of Morbid Anatomy*. It is to be issued in four parts, one of which has appeared. This work is especially valuable on account of its beautifully colored drawings, and we bespeak for it, what it should not fail to have, a welcome reception by the profession in the West. It is printed for subscribers only. Messrs. Desilver & Burr, Cincinnati, are the general agents.

